# Gene Classification and Mining of Molecular Markers Useful in Red Clover (*Trifolium pratense*) Breeding

**DOI:** 10.3389/fpls.2017.00367

**Published:** 2017-03-22

**Authors:** Jan Ištvánek, Jana Dluhošová, Petr Dluhoš, Lenka Pátková, Jan Nedělník, Jana Řepková

**Affiliations:** ^1^Department of Experimental Biology, Faculty of Science, Masaryk UniversityBrno, Czechia; ^2^Department of Psychiatry, University Hospital Brno and Masaryk UniversityBrno, Czechia; ^3^Agricultural Research Ltd.Troubsko, Czechia

**Keywords:** biosynthetic pathways, genetic diversity, sequencing, SNP, specific genes, SSR

## Abstract

Red clover (*Trifolium pratense*) is an important forage plant worldwide. This study was directed to broadening current knowledge of red clover's coding regions and enhancing its utilization in practice by specific reanalysis of previously published assembly. A total of 42,996 genes were characterized using Illumina paired-end sequencing after manual revision of Blast2GO annotation. Genes were classified into metabolic and biosynthetic pathways in response to biological processes, with 7,517 genes being assigned to specific pathways. Moreover, 17,727 enzymatic nodes in all pathways were described. We identified 6,749 potential microsatellite loci in red clover coding sequences, and we characterized 4,005 potential simple sequence repeat (SSR) markers as generating polymerase chain reaction products preferentially within 100–350 bp. Marker density of 1 SSR marker per 12.39 kbp was achieved. Aligning reads against predicted coding sequences resulted in the identification of 343,027 single nucleotide polymorphism (SNP) markers, providing marker density of one SNP marker per 144.6 bp. Altogether, 95 SSRs in coding sequences were analyzed for 50 red clover varieties and a collection of 22 highly polymorphic SSRs with pooled polymorphism information content >0.9 was generated, thus obtaining primer pairs for application to diversity studies in *T. pratense*. A set of 8,623 genome-wide distributed SNPs was developed and used for polymorphism evaluation in individual plants. The polymorphic information content ranged from 0 to 0.375. Temperature switch PCR was successfully used in single-marker SNP genotyping for targeted coding sequences and for heterozygosity or homozygosity confirmation in validated five loci. Predicted large sets of SSRs and SNPs throughout the genome are key to rapidly implementing genome-based breeding approaches, for identifying genes underlying key traits, and for genome-wide association studies. Detailed knowledge of genetic relationships among breeding material can also be useful for breeders in planning crosses or for plant variety protection. Single-marker assays are useful for diagnostic applications.

## Introduction

Fabaceae is among the most studied of plant families. The third-largest plant family, it includes many food and industrial plants and stands second only to Poaceae among the most important plant families from economic and nutritional perspectives (Graham and Vance, 2003). This importance results not only from the species' economic and nutritive values, but also from their unique capability for fixing atmospheric nitrogen. In the past decade, the extent of genomic information available on legumes has been broadened substantially. Such model species as *Medicago truncatula* Gaertn. (Young et al., 2011) and *Lotus japonicus* L. (Sato et al., 2008), as well as the crops soybean (*Glycine max* [L.] Merrill.; Schmutz et al., 2010), pigeon pea (*Cajanus cajan* [L.] Millsp; Varshney et al., 2012), and chickpea (*Cicer arietinum* L.; Varshney et al., 2013) have been sequenced. Several other sequencing projects are under way which encompass a broad range of agronomically and horticulturally important plants (www.phytozome.net).

Red clover (*Trifolium pratense* L.) belongs to the tribe Trifolieae, together with another 240 annual and perennial herb species, both wild and cultivated. It is an important forage plant worldwide, serving as a temporary cover crop or manure crop as well as for silage production and grazing. Like other legumes, it is capable of fixing atmospheric nitrogen via symbiosis with *Rhizobium leguminosarum* bv. *trifolii* (Sprent, 2009). Its breeding and related research have been complicated, however, by the species' outcrossing nature with gametophytic self-incompatibility. The resulting heterozygosity has hampered intensive genetic and genomic analysis. Nonetheless, with the rising availability of sequencing technology, red clover has been a target of several genomic studies in recent years.

Red clover's nuclear genome is divided into seven chromosomes (*x* = 7) with size estimated to be 418 Mbp (1C = 0.43 pg; Vižintin et al., 2006). The first consensus high-density linkage map contained 1,414 simple sequence repeats (SSRs), 181 amplified fragment length polymorphisms, and 228 restriction fragment length polymorphisms (Isobe et al., 2009). The structure of the red clover genome has been investigated using fluorescence *in situ* hybridization (Sato et al., 2005; Kataoka et al., 2012). The genome also has been compared with those of related species (white clover, *M. truncatula* and *L. japonicus*) using DNA markers (Isobe et al., 2012). DNA markers, too, can be used in various research and practical approaches. For example, two studies used DNA markers to identify quantitative trait loci (QTLs) related to persistence (Herrmann et al., 2008), disease resistance, and winter hardiness (Klimenko et al., 2010) in full sib mapping families. Recently, great insight into red clover genomics has been achieved through application of next-generation sequencing (NGS) technology. Both whole-genome sequencing (WGS; Ištvánek et al., 2014; De Vega et al., 2015) and RNA sequencing (Yates et al., 2014) have been carried out in red clover. While WGS focused on describing red clover's genome, RNA sequencing described transcriptome differences in conditions of drought stress. Concurrently, studies of both types identified a great number of DNA markers which can be of great value in practical applications.

As a consequence of the outcrossing, both natural ecotypes and varieties that may be morphologically similar are likely to be highly heterogeneous genetically. Strategies for genetic diversity analysis based on DNA profiling must address this issue and enable quantification of variation within and among populations. Evaluation of genetic variation for outcrossing forage species is important for the processes of cultivar identification and seed purity analysis, ecological analysis of pasture populations, and selection of genetically divergent parents for genetic mapping studies (Forster et al., 2001). The genetic divergence of some genotypes ensures a high level of genetic polymorphism in crosses. Breeding methods for cross-pollinated forage crops, including red clover, require strategies for genotyping. Genetic markers assaying variation in transcribed regions of genes with known functions will be useful for developing trait-linked markers. NGS has shown great potential for large-scale production of functional genes and molecular markers at the whole-genome level, especially in non-model organisms. Two important tasks for NGS are identifying expression patterns in biochemical processes and classifying genes into specific pathways. Legumes can produce more secondary metabolites (especially cyanogenic glucosides, glucosinolates, amines, and alkaloids) than can other plants which are not nitrogen fixers. Most secondary metabolites exhibit some biological, pharmacological, or toxicological activity (Teuscher and Lindequist, 2010; Wink, 2013). In this respect, the Fabaceae are distinguished by isoflavones, which function as antioxidants, phytoestrogens, and antimicrobial compounds. The benefits of protecting plant proteins from degradation in the rumen by means of polyphenol oxidases (PPOs) have been established in some fodder crops, and red clover contains PPOs in significant quantities (Jones et al., 1995; Jakešová et al., 2015). There is also an increasing need to develop molecular markers for resistance genes or components relating to nitrogen fixation.

Based on previously published genome assembly (Ištvánek et al., 2014), this study aims to elucidate red clover genes involved within complex biosynthetic pathways in response to biological processes. Special attention is given to specific secondary metabolites inasmuch as they can significantly influence the final variety's breeding strategy and purpose. Gene-specific SSR and single nucleotide polymorphism (SNP) markers are reported and described with a view to enhancing marker-assisted breeding in outcrossing species of red clover. Finally, we developed and validated sets of polymorphic microsatellites and SNPs for the analysis of genetic relationships among red clover varieties and individuals. The findings of this study can be useful in investigating genetic diversity and red clover breeding. These markers will contribute to enriching the current reference red clover map, generating more informative genetic and genomic tools, and enabling genome synteny analysis.

## Materials and methods

### Sequencing and gene annotation

Sequencing, *de novo* assembly, gene prediction, and initial annotation is described in Ištvánek et al. (2014). Whole Genome Shotgun project has been deposited at DDBJ/ENA/GenBank under the accession ASHM00000000. The version used in this paperis ASHM01000000. Detailed inspection of gene annotation was completed manually. Protein-encoding genes were classified into functional categories according to Gene Ontology (GO) annotation, and the results were summarized in plant GOslim functional categories. Each gene was aligned against KEGG (release 67.1) proteins, and the pathway in which that gene might be involved was determined. Genes were also sorted by the pathways.

### Comparison of *Trifolium* genes with model legumes

Homologous gene sequences were analyzed among red clover, *M. truncatula*, and *G. max*. Predicted genes in these species were mapped and searched for homology on the basis of red clover (DDBJ/EMBL/NCBI accession numbers: LT555306.1–LT555312.1; De Vega et al., 2015), *M. truncatula*, and *G. max* chromosome sequences. The best hits from a TBLASTX (*e* ≤ 1e^−15^) search with at least 70% identity were mapped on these chromosomes and gene densities for every 100 kbp were counted. Homologous sequences for these species were determined using the best hits from a reciprocal TBLASTX search. Circos software (Krzywinski et al., 2009) was used to visualize the top half hits of the data through a circular concentric ideograms layout. Gene densities were displayed as histogram plots and homologous sequences for the three species as lines.

### DNA marker prediction

SSR Locator (da Maia et al., 2008) was used to mine SSRs in the red clover genes as well as for primer design. Uniform melting temperature at T_m_ = 55°C was set for all predicted SSR sites, which were defined as a monomer occurring at least 12 times, a dimer occurring at least 6 times, tri- and tetramers occurring at least 4 times, and penta- and hexamers occurring at least 3 times. The number of polymerase chain reaction (PCR) products was predicted for each primer pair.

Probable SNPs in genes were discovered by aligning Tatra reads onto predicted genes using bwa v0.7.5 (Li and Durbin, 2010). Samtools v0.1.19 (Li et al., 2009) and Picard v1.80 (http://broadinstitute.github.io/picard/) were used in subsequent steps of marking PCR duplicates, sorting, and indexing. GATK v2.7 (https://www.broadinstitute.org/gatk/) was used to remap the reads near the InDels, recalibrate base quality scores, and identify SNPs. Only sites with SNP calling quality scores of 30 or higher and with read depths of at least 10 reads were marked as high-quality SNPs. For subsequent filtration, custom perl scripts were used.

### DNA marker validation

SSR marker validation was carried out for 50 red clover varieties, 34 of which were Czech varieties and 16 from other countries (Table 1). SNP marker validation was performed for 5 varieties (Amos, Fresko, Start, Tatra, Tempus). Leaves were collected from plants 30 days old which had been grown in a greenhouse. For SSR analysis, genomic DNA was isolated from 16 pooled plants per variety from ~1 g of young leaves using the modified protocol by Dellaporta et al. (1983). Concentration and purity of extracted genomic DNA was assessed using NanoDrop (Thermo Scientific, Waltham, MA, USA).

**Table 1:** **List of red clover varieties and their characterization**.

ECN^*^	**Variety**	**Ploidy**	**Origin**
13T0200096	Agil	2x	CZ
13T0200080	Bonus	2x	CZ
13T0200097	Brisk	2x	CZ
13T0200034	Chlumecky	2x	CZ
13T0230104	Concorde	2x	US
13T0200018	Essex broad red	2x	GB
13T0200081	Garant	2x	CZ
13T0200357	Gibridnij pozdnespelyj	2x	SU
13T0200492	Grasslands hamua	2x	NZ
13T0230094	Makimidori	2x	JP
13T0200625	Nemaro	2x	DE
13T0200568	Parka	2x	PL
13T0200056	Pavo	2x	CH
13T0200496	Radan	2x	CZ
13T0200127	Respect	2x	CZ
13T0200029	Slavin	2x	CZ
13T0200030	Slavoj	2x	CZ
13T0230101	Spurt	2x	CZ
13T0200319	Start	2x	CZ
13T0230006	Suez	2x	CZ
13T0200598	Tabor	2x	CZ
13T0200039	Trubadur	2x	CZ
13T0200020	Van	2x	CZ
13T0230090	Vendelin	2x	CZ
13T0200600	Vltavin	2x	CZ
13T0230095	Walter	2x	CA
13T0230023	Amos	4x	CZ
13T0200058	Astur	4x	CH
13T0200088	Atlantis	4x	DE
13T0230132	Beskyd	4x	CZ
13T0230024	Bivoj	4x	CZ
13T0230021	Blizard	4x	CZ
13T0230034	Cyklon	4x	CZ
13T0230133	Dolina	4x	CZ
13T0230039	Dolly	4x	CZ
13T0230145	Fresko	4x	CZ
13T0200571	Hungarotetra	4x	HU
13T0200329	Kvarta	4x	CZ
13T0200486	Lossam	4x	FR
13T0230026	Margot	4x	SK
13T0200476	Radegast	4x	CZ
13T0230110	Rezista	4x	CZ
13T0230120	Sigord	4x	SK
13T0230134	Sprint	4x	CZ
13T0200327	Tatra	4x	CZ
13T0200538	Tempus	4x	CZ
13T0230114	Titus	4x	CZ
13T0200460	Triton	4x	SE
13T0200597	Vesna	4x	CZ
13T0200636	Vulkan	4x	CZ

* *National accession number—GeneBank of Crop Research Institute Ltd., Prague-Ruzyně, Czech Republic; CA, Canada; CH, Switzerland; CZ, Czech Republic; DE, Germany; FR, France; HU, Hungary; JP, Japan; NZ, New Zealand; PL, Poland; SE, Sweden; SK, Slovakia; SU, Soviet Union; US, United States*.

To validate SSRs, we randomly selected 96 SSR loci in coding sequences. SSR primers (Table S1) were predicted by SSR Locator. Validations of predicted SSRs were performed via PCR and electrophoretic separation. PCRs were carried out in a volume of 10 μl with 1x reaction buffer, 0.2 mM of each dNTP (10 mM; Sigma-Aldrich, Steinheim, Germany), 10 pmol of each primer, 0.5 U of Go*Taq*® polymerase (Promega, Madison, WI, USA), and 30 ng of genomic DNA template.

Cycling conditions were set as follows: a preliminary step at 94°C for 3 min, 58°C for 1 min, 72°C for 1 min; 30 cycles of 94°C for 30 s, 58°C for 30 s, and 72°C for 30 s; and an elongation step at 72°C for 5 min. PCR-amplified fragments were separated by electrophoresis on either a 3% agarose gel or a 10% polyacrylamide gel and visualized by ethidium bromide staining.

The validation of predicted SNP variants was performed by SNP array (Arrayit Corporation, CA, USA) with 8,623 genome-wide distributed SNPs. We examined intra-variety genetic heterogeneity using SNP genotyping for a set of 20 DNA samples of individual red clover plants of the variety Tatra. Probes, 15-mer oligonucleotides, were designed with the SNP at the center position without overlap (two probes per SNP; Table S2). The fluorescent dyes Alexa Fluor® 555 and Alexa Fluor® 647 (Invitrogen, CA, USA) were used for labeling. Capture agents were printed into 1–48 microarrays per 25 × 76 mm glass substrate slide, each probe 3 times. Hybridization was performed with genomic DNA isolated from individual plants using the modified protocol by Dellaporta et al. (1983). Variance stabilizing normalization was used to evaluate fluorescence intensities of the reference and alternative alleles by RStudio software (RStudio Team, 2015) with limma package (Ritchie et al., 2015) and the log2 intensity data were processed.

Single-marker SNP polymorphisms were also validated by the modified technique of temperature switch PCR (Tabone et al., 2009) whereby two PCRs were carried out for each locus, one for a reference (*R*) allele and the other for an alternative (*A*) allele. Due to this technique's specific requirements, only SNP sites having no other SNP in their vicinity for 30 bp were included within the validation. Genomic DNA was isolated from 100 mg of leaves of individual plants using the CTAB method (Rogers and Bendich, 1989). SNP validation was performed for five candidate SNPs from Tatra coding sequences used as a reference. Primers (Table S3) for SNPs were predicted using Primer3 and OligoCalc. For an *R* allele, two primer pairs were used (LS—locus specific and NLS—nested locus specific) with different melting temperatures. PCR amplification using these four primers provides 4 amplicons, while only the amplicon emerging from the NLS primers (with NLS_F primer directly binding to the SNP position) confirms the presence of an *R* allele in a selected sample. For reliable detection of an *A* allele, we performed a simplified PCR reaction with two primers, a forward LS primer and a reverse primer (labeled as reverse primer SNP; SNP_R) which binds with its 3′ end to a SNP nucleotide. The presence of a PCR product of a predicted length confirms the existence of an *A* allele. All expected products with their predicted lengths are listed in Table S4. The PCR components used for both PCRs were the same as for the SSR validation, with minor modifications: for detection of the *R* allele, 5 pmol of each NLS primer and 1 pmol of each LS primer were used. Also, PCR was enriched with 1% *bovine* serum albumin. To detect the *A* allele, 5 pmol of both primers were used. Both PCRs used Go*Taq*® polymerase in a concentration of 0.25 U. Cycling conditions for the *R* allele were used according to Tabone et al. (2009). Cycling conditions for the *A* allele were shortened to a denaturation step at 95°C for 5 min; 30 cycles of 95°C for 30 s, 62°C for 30 s, and 72°C for 30 s; then 72°C for 5 min as a final elongation step. PCR-amplified fragments were separated by electrophoresis on a 10% polyacrylamide gel and visualized by ethidium bromide staining.

### Polymorphism evaluation

Evaluation of individual polymorphic fragments was inferred from agarose or polyacrylamide gels with separated PCR-amplified fragments and was performed for each SSR marker manually. Pooled polymorphism information content (pPIC) was then calculated for each SSR marker, expressing the probability of detecting a polymorphism between genotypes of two randomly drawn red clover varieties. pPIC was calculated for all 50 red clover varieties. Calculations were performed for each SSR marker *m* separately as follows:

1. Genotypes were divided into two groups (*G*_1_ and *G*_0_) according to the presence/absence of any PCR-amplified product for the marker *m* (i.e., *G*_1_ contained all genotypes with at least one PCR-amplified product and *G*_0_ contained the rest).
2. pPIC for the subgroup G_1_ was calculated as:
  $$pPIC_{1}=1-\underset{i\;=\;1}{\prod^{n}}\left(1-2f_{i}\left(1-f_{i}\right)\right),$$
  where *i* is one particular band from *n* possible bands of this marker; Π is the product operator, i.e., the symbol denoting product of a sequence in a similar manner as ∑ denotes summation; and *f*_*i*_ is the frequency of the *i*-th band among all genotypes in *G*_1_. *pPIC*_1_ is thus the probability that the marker *m* can distinguish any two random genotypes whose probabilities for the presence of each band come from the same distribution as in the sample *G*_1_.
3. pPIC for the subgroup *G*_0_ was *pPIC*_0_ = 0, because the genotypes in *G*_0_ cannot be distinguished from each other (they did not have any PCR-amplified products).
4. pPIC for comparison of two genotypes, one from each group, was *pPIC*_1 × 0_ = 1, because any genotype from *G*_1_ can be distinguished from any *G*_0_ genotype by having at least one product.
5. Overall pPIC for the marker *m* was then calculated by weighting the values acquired in previous steps according to the proportionate sizes of *G*_1_ and *G*_0_:
  $$\begin{array}{l} pPIC=p_{1}^{2}\;\cdot \;pPIC_{1}\;+\;2p_{1}p_{0}\;\cdot \;pPIC_{1\;\times \;0}\;+\;p_{0}^{2}\;\cdot \;pPIC_{0}\\ \;=p_{1}^{2}\;\cdot \;pPIC_{1}\;+\;2p_{1}p_{0}1\;+\;p_{0}^{2}\;\cdot \;0\\ \;=p_{1}^{2}\;\cdot \;pPIC_{1}+2p_{1}p_{0}, \end{array}$$
  where $p_{1}=\frac{N_{1}}{N_{1}\;+\;N_{0}}$, $p_{0}=\frac{N_{0}}{N_{1}\;+\;N_{0}}$ are proportions of genotypes in the two groups *G*_1_ and *G*_0_ (*N*_1_ and *N*_0_ are counts of genotypes in *G*_1_ and *G*_0_ for the marker *m*).

The polymorphic information content (PIC) of the SNP loci was calculated according to Botstein et al. (1980).

### Phylogenetic analysis

The similarity between each pair of *T. pratense* varieties was assessed according to the presence or absence of individual separated PCR-amplified fragments using the Jaccard (1901) and Sørensen–Dice (Dice, 1945; Sørenson, 1948) indices for each SSR marker. The Jaccard and Sørensen–Dice indices were calculated as n_xy_/(n_x_ + n_y_ – n_xy_) and 2n_xy_/(n_x_ + n_y_), respectively, where n_xy_ represents the number of bands which are present simultaneously in both compared varieties, n_x_ represents the number of all bands of one of the compared varieties, and n_y_ represents the number of all bands of the other compared variety.

Separately for each of the indices, these coefficients of similarity were used for calculating a pairwise distance matrix for each marker, where the distance between two selected varieties was computed as 1—the corresponding similarity coefficient. Finally, an averaged distance matrix was created by averaging distance matrices of all markers. Thus, two pairwise distance matrices—one based on the Jaccard index and one on the Sørensen–Dice index—were created, describing the averaged dissimilarity between each pair of red clover varieties. Two phylogenetic trees based on the averaged distance matrices were calculated in MATLAB (version R2015a, http://www.mathworks.com) using the unweighted pair group method with arithmetic mean (UPGMA) clustering method then manually edited and visualized in FigTree (version 1.4.2, http://tree.bio.ed.ac.uk/software/figtree/).

## Results

### Sequencing and gene annotation

**Figure 1:**
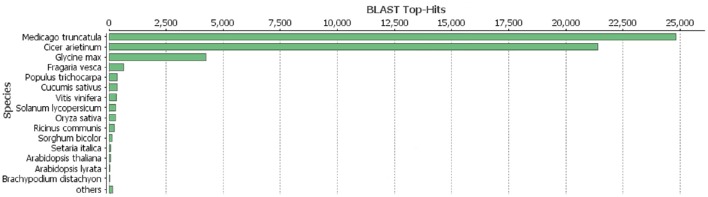
**BLASTP top-hits distribution of red clover annotated genes with RefSeq database**.

As described in Ištvánek et al. (2014), 243.6 million reads were obtained by sequencing. After filtering out low-quality reads and sequencing adapter relics, genome coverage of ~55.4x was achieved. A total of 64,761 genes were predicted in red clover (Ištvánek et al., 2014) and after manual revision of Blast2GO annotation, 42,996 genes were characterized. These included 1,316 genes related to repetitive elements (Table 2). One of the main annotation steps was based on finding the sequence homology with accessions in the RefSeq database (BLASTP search). The results of this part are summarized in Figure 1 in the form of Blast Top-Hits, showing the degree of relationship to other sequenced plant model species. All predicted genes with their annotations are displayed in Table S5.

**Table 2:** **Red clover gene characteristics**.

**PREDICTED GENES**	
Number of genes	64,761
Number of exons	202,783
Number of introns	160,364
Mean number of exons per gene	3.1
Mean number of introns per gene	2.5
Mean gene length (bp)	1,480.3
Mean exon length (bp)	244.6
Mean intron length (bp)	288.5
Single-exon genes	11,559 (17.8%)
**ANNOTATED GENES**	
Number of genes	42,996
Number of exons	160,421
Number of introns	131,968
Mean number of exons per gene	3.7
Mean number of introns per gene	3.1
Mean gene length (bp)	1,818.1
Mean exon length (bp)	244.9
Mean intron length (bp)	294.6
Single-exon genes	6,854 (15.9%)
**GENES WITHOUT ANNOTATION**	
Number of genes	21,765
Number of exons	42,362
Number of introns	28,396
Mean number of exons per gene	1.9
Mean number of introns per gene	1.3
Mean gene length (bp)	813.0
Mean exon length (bp)	243.3
Mean intron length (bp)	260.1
Single-exon genes	4,705 (21.6%)
**GENES RELATED TO REPETITIVE ELEMENTS**	
Number of genes	1,316
Number of exons	2,360
Number of introns	1,382
Mean number of exons per gene	1.8
Mean number of introns per gene	1.1
Mean gene length (bp)	1,359.0
Mean exon length (bp)	655.6
Mean intron length (bp)	174.6
Single-exon genes	301 (22.9%)

**Figure 2:**
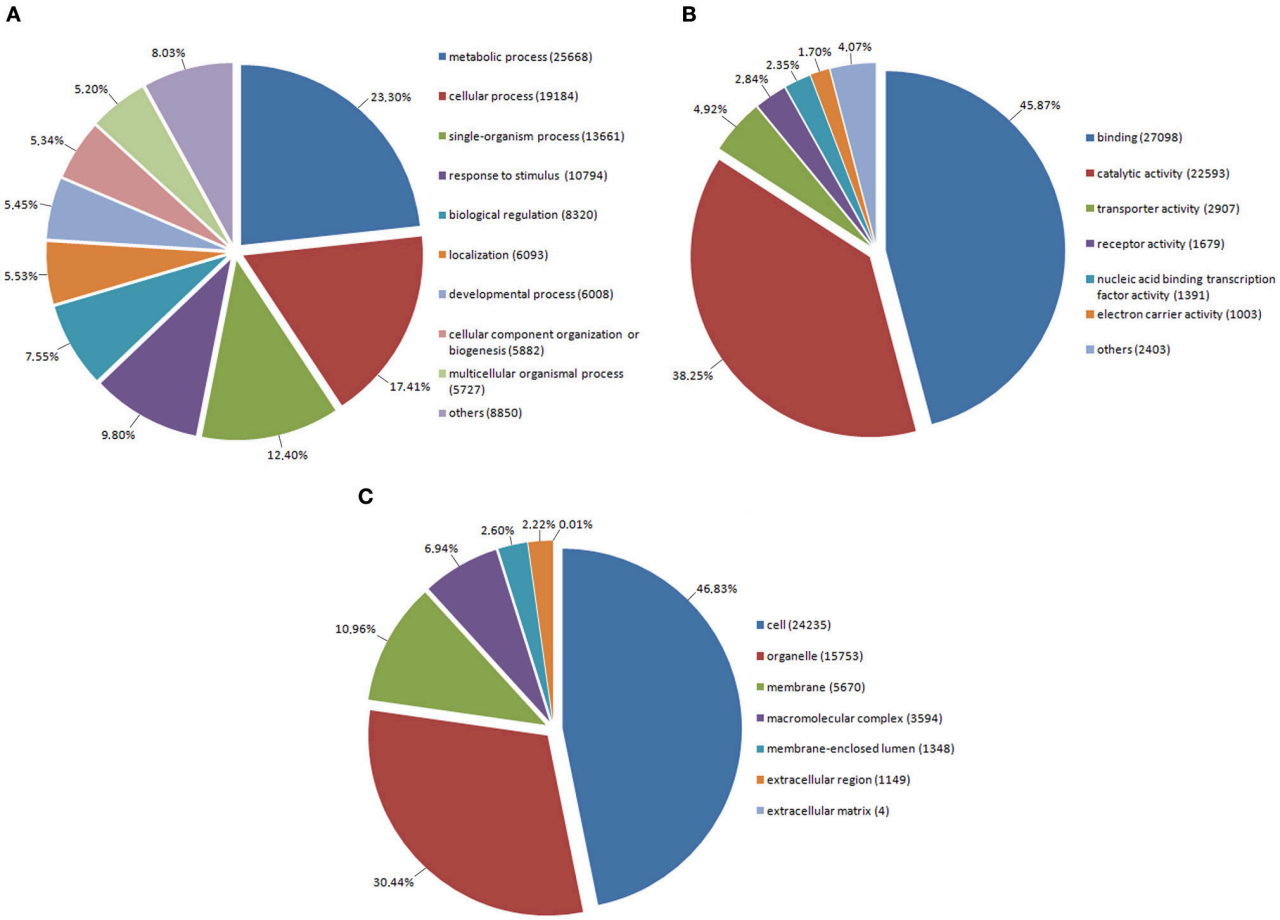
**Assignment of red clover genes into Gene Ontology (GO) terms in level 2 belonging to: (A)** Biological Process, **(B)** Molecular Function, **(C)** Cellular Component.

Annotated genes were assigned to appropriate biological process, molecular function, and cell component subclasses based on their annotation (Figure 2). Within the sequences associated with biological processes, GO terms associated with primary and secondary metabolism were the most prevalent. In this respect, primary metabolites are known to be essential for plant survival while secondary metabolites play important roles in plant protection and have a broad spectrum of utilization. We also found genes associated with the GO term “response to stimulus” to occur very frequently. This category includes mainly genes involved in responses to stress, biotic stress, and endogenous as well as extracellular stimuli. In molecular function, almost one-half of genes are associated with the GO term “binding,” in which the binding functions of nucleotides or DNA form the majority. “Catalytic activity,” as the second most prevalent term, comprises enzymatic activities of kinases, hydrolases, nucleases, etc. In cellular component, the most frequent GO terms were associated with functions within the plant cell, organelles (plastids and mitochondria), and plasma membrane. In short, these relate to the main cellular compartments of plant cells.

**Figure 3:**
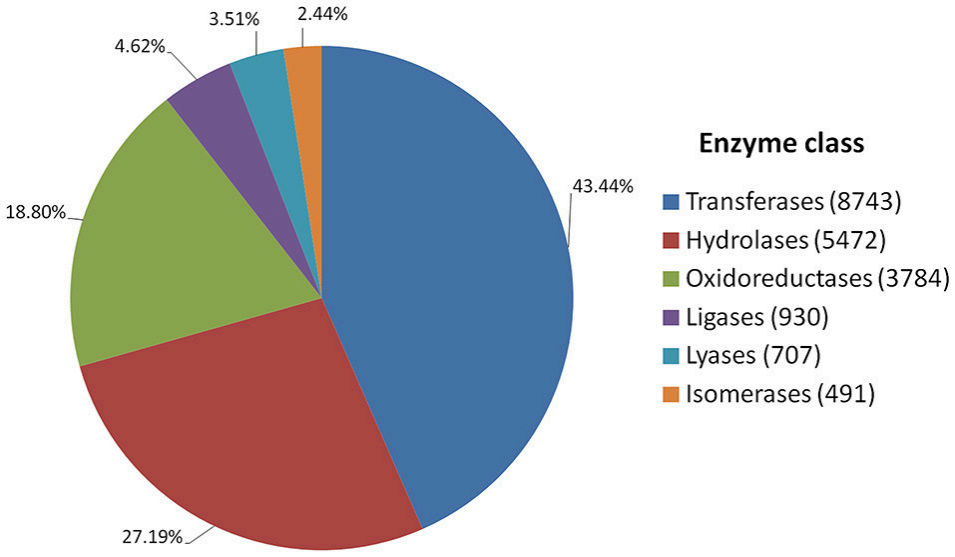
**Catalytic activity distribution in red clover annotated genes**.

Annotated genes were also classified into metabolic and biosynthetic pathways. A total of 7,517 genes were characterized and assigned to specific pathways. Because many genes figure in multiple biosynthetic or metabolic pathways, a total of 17,727 enzymatic nodes were described in all pathways. Among the largest metabolic pathways (each involving more than 1,000 genes) were purine metabolism and starch and sucrose metabolism. The 20 largest biosynthetic pathways are summarized in Table 3. Table S6 presents a complete list of genes assigned to specific metabolic and biosynthetic pathways. Each enzyme was also assigned to one of the main enzyme classes (Figure 3). In red clover, almost one-half of enzymes belong to transferases (43.44%), and more than 89% of enzymes consist of transferases, hydrolases, and oxidoreductases.

**Table 3:** **Twenty largest biosynthetic and metabolic pathways in red clover based on number of genes (enzymes) involved**.

**Pathway**	**Seqs. in pathway**
Purine metabolism	1,138
Starch and sucrose metabolism	1,053
Phenylalanine metabolism	622
Pentose and glucuronate interconversions	475
Phenylpropanoid biosynthesis	469
Thiamine metabolism	428
Pyrimidine metabolism	378
Glycerolipid metabolism	364
Cysteine and methionine metabolism	354
Galactose metabolism	328
Amino sugar and nucleotide sugar metabolism	323
Glycerophospholipid metabolism	304
T cell receptor signaling pathway	297
Glycolysis/Gluconeogenesis	284
Tyrosine metabolism	273
Phenylalanine, tyrosine and tryptophan biosynthesis	272
Flavonoid biosynthesis	257
Arginine and proline metabolism	253
Glutathione metabolism	242
Pyruvate metabolism	240

### Comparison of red clover genes with model legumes

**Figure 4:**
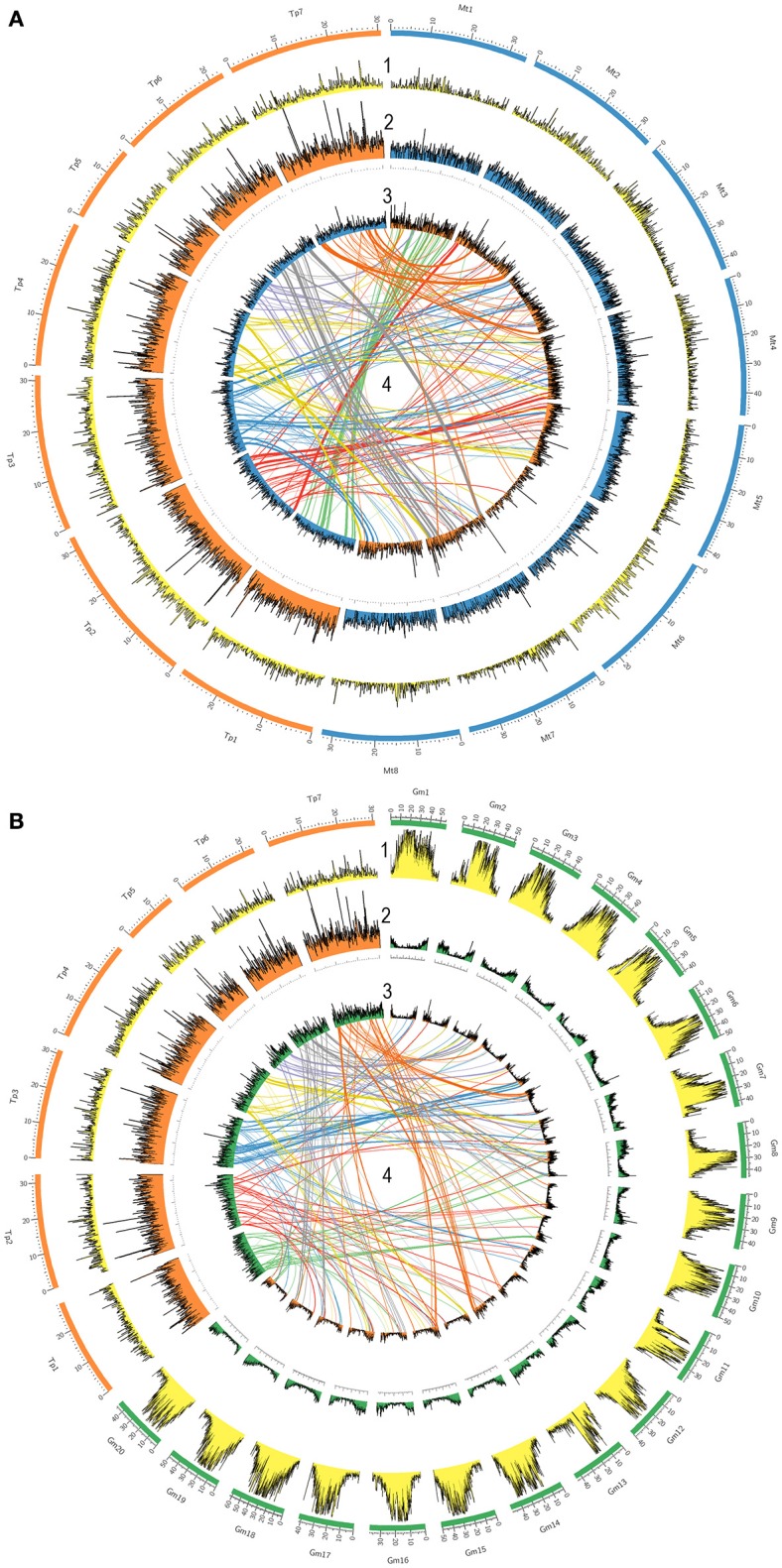
**Comparison of gene densities and genome structure in legume model species (A)** *M. truncatula* and **(B)** *G. max* with *T. pratense*. The 7 *T. pratense* chromosomes (DDBJ/EMBL/NCBI accession numbers: LT555306.1 - LT555312.1) are shown in orange, 8 *M. truncatula* chromosomes in blue, and 20 *G. max* chromosomes in green in the outer circles. (1) First circles represent repetitive element densities relevant to each chromosome (yellow). Gene densities (by 100 kb windows) are displayed on each chromosome as follows: (2) gene density in *T. pratense* (orange), *M. truncatula* (blue), and *G. max* (green) on their own chromosomes; (3) relative gene densities of *T. pratense* on *M. truncatula* and *G. max* chromosomes mapped on the partner's chromosomes; (4) homologous sequences and synteny regions in *T. pratense* with *M. truncatula* and *T. pratense* with *G. max* (central lines; top half is colored).

A TBLASTX search was performed to evaluate the distribution of all red clover genes along recently published chromosomes of red clover, chromosomes of the model legume species *M. truncatula* (8 chromosomes), and chromosomes of *G. max* (20 chromosomes). The results were plotted using a window size of 100 kb through genomic sequences (Figure 4). Repetitive element and gene densities in each species were distributed along all chromosomes of *T. pratense, M. truncatula*, and *G. max*. Distribution patterns were similar in both *T. pratense* and *M. truncatula*. Under the specified criteria, 41,607 red clover genes were found to be homologs in comparison with *M. truncatula* and 32,737 genes were homologs with *G. max*. In *G. max*, the genes were concentrated in subtelomeric and telomeric regions. These are regions with low density of repetitive elements, unlike centromeric regions (Torales et al., 2013). This can be seen also in the central lines that show the distribution of homologous sequences to *M. truncatula, G. max*, and red clover. On the other hand, the gene densities in *M. truncatula* are the more balanced, with only a slight decrease in centromeric regions. Centromeric regions were also poorer for homologous sequences, for example in chromosome Mt6 and Mt8.

### Predicted DNA markers

**Figure 5:**
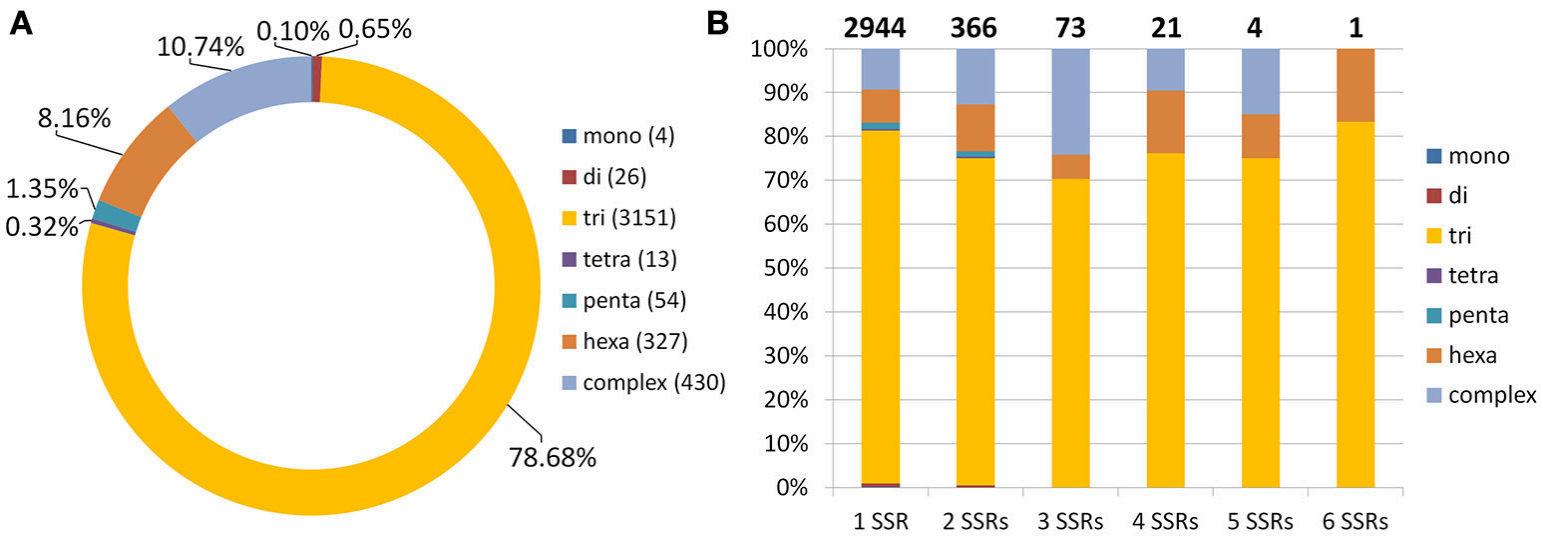
**Statistics for 4,005 predicted SSR markers in coding sequences of red clover. (A)** Basic motif frequencies in coding regions of red clover. **(B)** Frequencies of mono-, di-, tri-, tetra-, penta-, and hexamers plus complex SSR motifs in red clover genes containing one to six SSRs per locus. Total number of sequences containing depicted number of SSRs is shown above each column.

Using SSR Locator (da Maia et al., 2008), we identified 6,749 potential microsatellite loci in red clover coding sequences. For those with sufficient flanking sequences, we designed appropriate unique primers to generate PCR product preferentially within 100–350 bp. The resulting 4,005 (59.3%) potential SSR markers were characterized (Figure 5). Because 1,061 (26.5%) of these SSR markers occurred in an identical unique locus, it results that just 3,409 (5.3%) coding sequences possess at least one SSR marker. When the total length of coding sequences (49.6 Mbp) is taken into count, marker density of 1 SSR marker per 12.39 kbp was achieved. Especially noteworthy is that no SSR markers were found in the genes belonging to the isoflavonoid biosynthetic pathway, such as 2-dihydroflavonol reductase, chalcone synthase, and isoflavone synthase. All potential SSR markers are shown in Table S7.

As expected, the most frequently seen basic motif of microsatellite corresponded to trimeric repeat (78.68%), followed by complex (10.74%) and hexameric (8.16%) motifs (Figure 5). These motifs were also present mainly in loci with a single SSR marker. Complex motifs consisted mainly of two–five trimeric motifs, with only 55 (12.8%) exceptions containing also other motifs (mainly hexameric). Other motifs, such as dimeric and pentameric, were seen much less frequently. Only in 7 SSRs with complex motifs did the complex motif not contain a trimeric repeat.

SNPs were identified by aligning reads to predicted coding sequences. The analysis resulted in identification of 343,027 SNP markers, providing marker density of 1 SNP marker per 144.6 bp, meaning on average 5.3 SNP markers per gene. Of these SNPs, 290,905 (84.8%) SNPs were high quality. SNP markers were also divided between transitions and transversions based on the nature of the *A* allele. The majority (nearly two-thirds) of SNP markers were transitions. In addition, 4,065 (1.19%) of the identified SNP markers were multi-allelic, with more than one *A* allele. Table 4 presents a complete overview and statistics relating to SNP markers. Table S8 summarizes the complete list of SNP markers, including their positions and additional information.

**Table 4:** **Statistical overview of SNP markers predicted in red clover**.

**Transitions**			**Transversions**		
**SNP**	**Number**	**Prevalence %**	**SNP**	**Number**	**Prevalence %**
A <-> G	109,284	50.15	A <-> C	32,387	26.75
C <-> T	108,610	49.85	C <-> G	21,276	17.57
Total	217,894	63.52	G <-> T	29,231	24.14
			T <-> A	38,174	31.53
Multi-allelic SNP	4,065	1.19	Total	121,068	35.29

### Validation and polymorphism of predicted SSR markers, phylogenetic analysis

**Figure 6:**
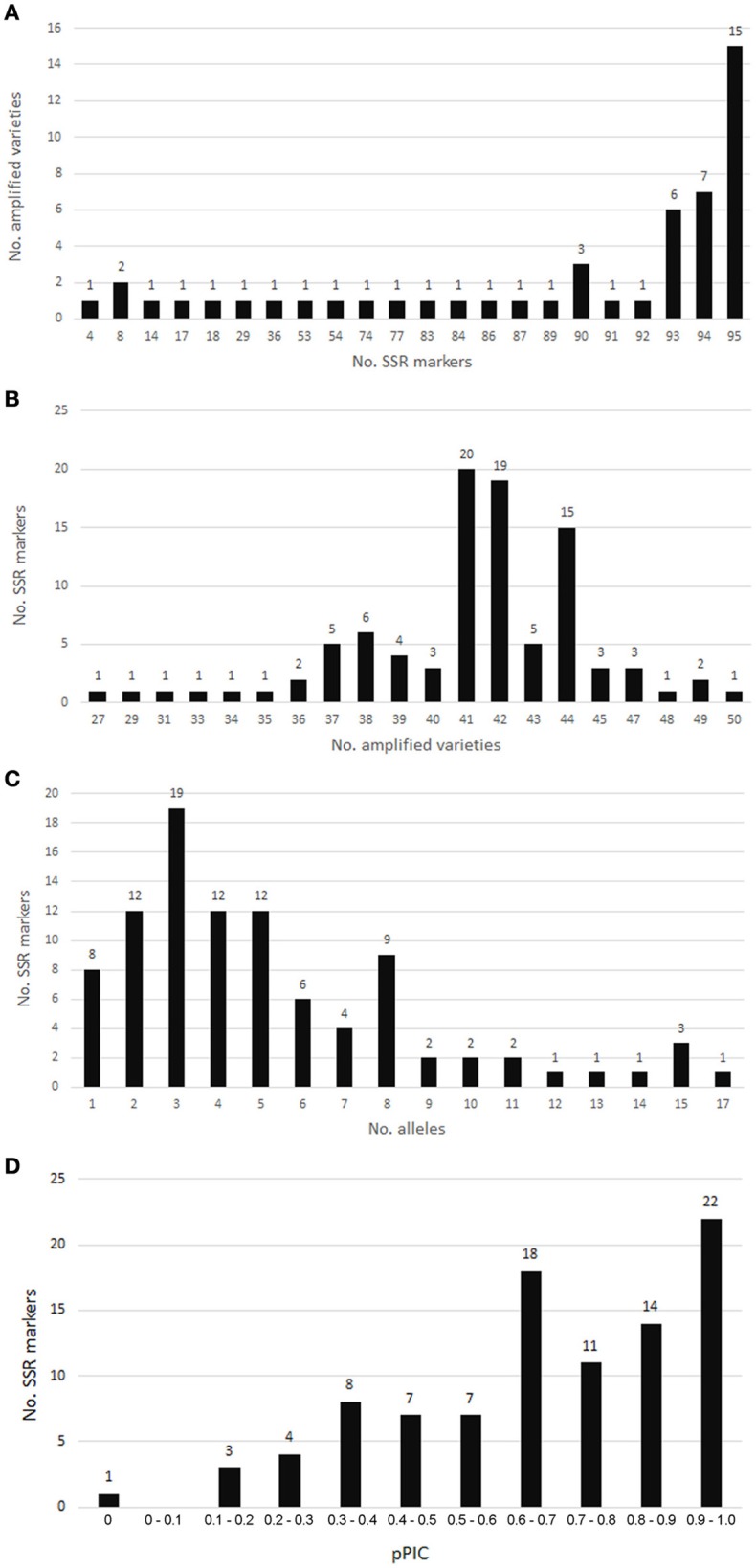
**SSR marker validation in red clover varieties. (A)** Numbers of SSRs amplified in analyzed varieties. **(B)** Numbers of varieties amplified for individual SSRs. **(C)** Allele number distribution for SSR markers validated in red clover varieties. **(D)** pPIC distribution for SSR markers validated in red clover varieties.

Of 96 chosen SSR loci, only the SSR locus SSR-TP_g53834.t1.cds1 was not amplified. Altogether, 95 SSRs were analyzed for 50 red clover varieties. SSR markers with a PCR product are summarized in Table S1. Single monomorphic SSR marker SSR-TP_g20700.t1.cds3 was amplified in all 50 varieties. The lowest number of amplified samples/varieties was 27 (Figure 6). Fifteen varieties gave a PCR product for all 95 SSRs, and the lowest number of markers (4) was amplified in a single variety (Radegast).

Allele number ranged from 1 to 17 (Table S1, Figure 6). The pPIC of these SSR loci ranged from 0 to 0.986 with a mean of 0.679 and median of 0.693 (Table S1). The highest diversity was determined for SSR loci with trinucleotide motifs and pPIC ranging from 0.180 to 0.986 (Table 5). Twenty-two SSRs out of 95 validated were highly polymorphic and with pPIC >0.9 while 72 SSRs showed pPIC >0.5 (Figure 6).

**Table 5:** **pPIC in validated red clover SSRs with different motifs**.

**Repeat type**	**Number of SSRs**	**pPIC**		
		**Range**	**Mean**	**SD**
Monomer	2	0.567–0.693	0.6297	0.0632
Dimer	2	0.875–0.895	0.8853	0.0102
Trimer	67	0.180–0.986	0.6929	0.2227
Hexamer	2	0.333–0.365	0.3488	0.0160
Complex	22	0–0.975	0.6514	0.2734

**Figure 7:**
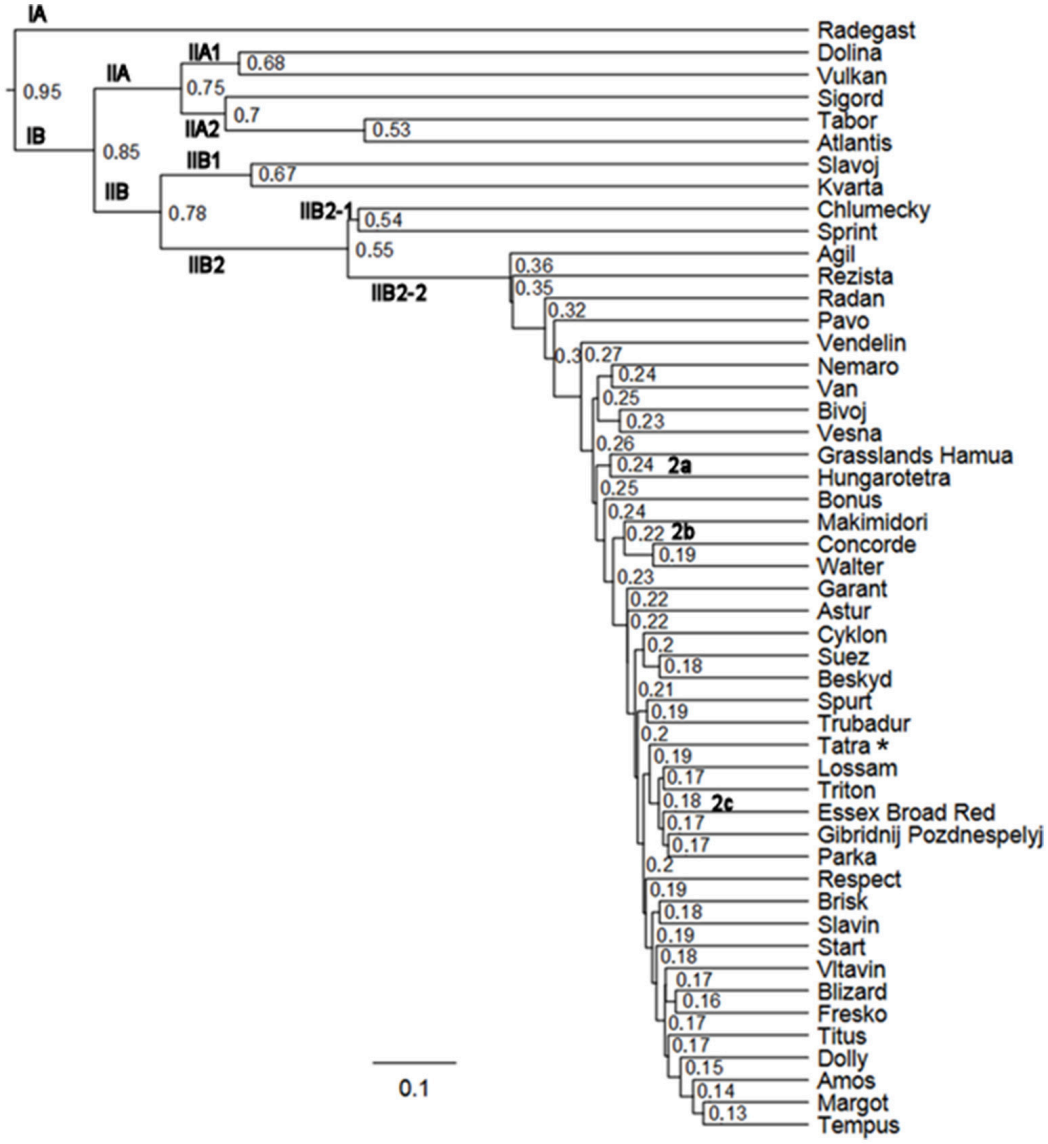
Study of red clover variety divergence using SSR markers (Sørensen-Dice index, ^*^reference genotype).

The similarity between individual varieties of *T. pratense* was assessed using the Sørensen–Dice and Jaccard indices (Figure 7, Figure S1). Cluster analysis grouped the 50 red clover varieties into two clusters. Sub-cluster IA consisted of the single variety Radegast (4x), developed from landraces that were well adapted locally, from breeding varieties (Slovensky podtatransky, Chlumecky, and Horal) and a later cross with the variety Weitetra. Sub-cluster IIA comprised a group of varieties whose genomes were enriched with genotypes of European origin: Dolina (4x), Vulkan (4x), and Sigord (4x) were developed by crosses of Czech, Polish, and German varieties; Tabor (2x) was developed by mass crosses of selected resistant plants belonging to 49 varieties; and Atlantis (4x) is of German origin. Cluster IIB1 consisted of two varieties: Slavoj (2x) was developed by the selection of genotypes, and Kvarta (4x; released 1974) was developed by polyploidy of landraces and the variety Chlumecky (2x), which itself was a component of the next sub-cluster IIB2-1. Chlumecky is the earliest red clover cultivar (released 1935) developed by individual plant selection from the landrace Cesky. Sprint (4x) was obtained after the polyploidy of four newly bred genotypes of European origin.

IIB2-2 was a large cluster of diploid and tetraploid Czech, European, and non-European varieties, reflecting that the varieties are often populations developed from genotypes with wide genetic variability, by targeted crosses, polycrosses, and topcrosses suitable for the selection of complex characters, with synteny of the selected genotypes. The sub-clusters of non-Czech origin varieties were as follows: IIB2-2a Grasslands Hamua from New Zealand and Hungarotetra from Hungary; IIB2-2b three varieties of non-European origin (Makimidori, Concorde, Walter); and IIB2-2c, five European varieties (Lossam, Triton, Essex Broad Red, Gibridnij Pozdnespelyj, Parka). In addition, Pavo and Astur were released in Switzerland, Nemaro, and Titus in Germany, and Vesna is of Czech origin but developed by crosses of diploid genotypes from Czech, French, Swiss, and German varieties with subsequent polyploidy by colchicine. Slovak and Swedish red clover genetic material was introgressed into the genome of Tatra, and Blizard was bred using non-Czech genotypes and recurrent phenotypic selection. Start, released in 1974, was used as a component for more recently bred varieties such as Garant, Cyklon, Spur, Trubadur, Dolly, and Tempus. The same cluster distribution was observed using the Sørensen–Dice and Jaccard indices, with only a few exceptions in cluster IIB2-2 (Figure 7, Figure S1), such as the sub-clusters Grasslands Hamua and Hungarotetra, Cyklon, Suez, Beskyd, Spur, and Trubadur.

### Validation of SNP markers and their polymorphism

We examined intra-variety genetic heterogeneity using genome-wide SNP genotyping. Five possible genotypes for two alleles per SNP (reference *R*, alternative *A*) were differentiated (*RRRR, RRRA, RRAA, RAAA, AAAA*) for tetraploid plants. Our analysis revealed 8,607 polymorphic SNP markers with PIC ranging from 0.024 to 0.375 with a mean of 0.338 and median of 0.355 (Table S9).

**Figure 8:**
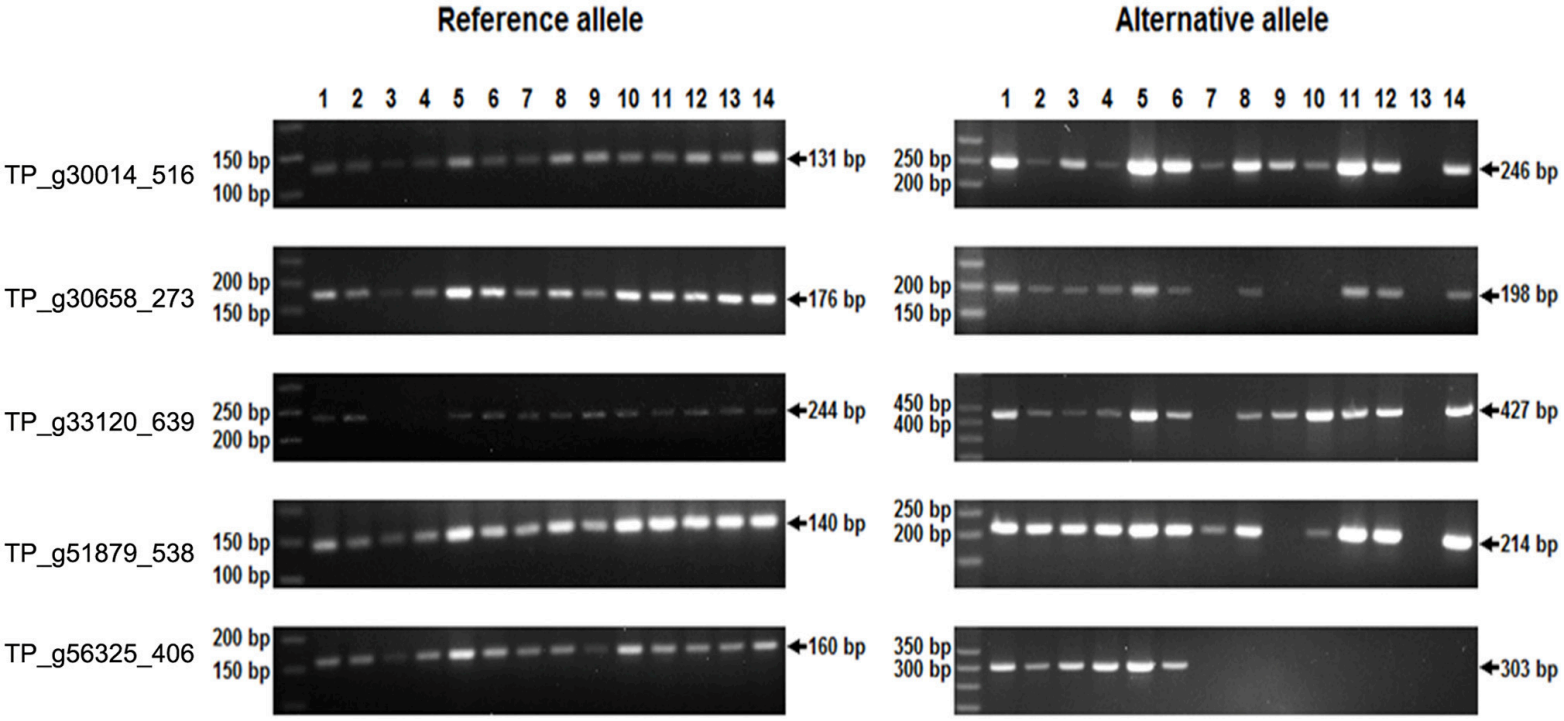
**SNP marker validation in red clover individuals by temperature switch method**.

Single-marker polymorphism was successfully validated and confirmed in five SNP loci and homozygosity/heterozygosity was determined in 14 particular plants, 7 of which were of variety Tatra, 4 of variety Tempus, and 1 plant of each Start, Amos, and Fresco. The majority of those plants analyzed were heterozygous in the tested loci. Homozygosity for the *R* allele was detected in one plant (Tempus) in TP_g30014_516, four plants (Tatra, Amos, Fresco, Tempus) in TP_g30658_273, two plants (Tatra, Tempus) in TP_g33120_639, two plants (Amos, Tempus) in TP_g51879_538, and eight plants (Tatra, Start, Amos, Fresco, and all Tempus) in TP_g56325_406. Two homozygotes of Tatra were detected for the *A* allele in TP_g33120_639 (Figure 8). All amplified fragments corresponded to those predicted.

## Discussion

### *T. pratense* genes

The number of annotated genes in this study is higher than the number of genes identified recently by RNA sequencing (34,534 genes; Yates et al., 2014), but it is very close to the number from other WGS (40,868 genes; De Vega et al., 2015). On the basis of improved annotation of red clover genes, genes were classified into biosynthetic and metabolic pathways and key enzymes were identified. We have found 1,138 genes involved in purine metabolism, which is the fundamental pathway for plant growth and development (Zrenner et al., 2006). This pathway is associated with DNA synthesis, energy sources, and synthesis of many primary and secondary metabolic products (Stasolla et al., 2003). More than 1,000 genes are also involved in starch and sucrose metabolism, which is one of the most important pathways regarding energy sources in plants. Moreover, with 257 genes involved, biosynthesis of flavonoids is among the largest metabolic and biosynthetic pathways. These genes are of particular interest to red clover breeders inasmuch as flavonoid biosynthesis is associated with isoflavonoid content in the plant. Because they are known plant estrogen analogs, high levels of isoflavonoids are undesirable in forage varieties (Adams, 1995) but these are required in varieties used in pharmaceutics (Park and Weaver, 2012).

Based on the distribution of homologous sequences among red clover, *M. truncatula*, and *G. max*, very similar patterns in gene distribution are present in red clover and *M. truncatula*. In contrast to *G. max*, gene density is rather uniform along entire chromosomes. Occasional spikes in density of red clover genes show clusters of numerous gene families, such as genes of resistance. As previously described (Kulikova et al., 2004; Isobe et al., 2012; Torales et al., 2013), *M. truncatula* had fewer homologous genes located on its chromosome 6 due to the abundance of heterochromatic sites and retroviral elements scattered throughout its arms. This was in contrast to red clover, where no such decrease was observed. The density of repetitive content along the chromosomes also was inspected and this provided very similar results. Also visible, however, is increased content of repetitive elements in red clover (Ištvánek et al., 2014) compared to *M. truncatula* (Young et al., 2011). Orthologous loci were connected and compared among species visualizing syntenic loci and rearrangements of genome structure. During speciation, red clover clearly underwent complex genome restructuring, possibly associated with reduction of the basic chromosome number from eight to seven. Results supporting this hypothesis were also found in a comparison among red clover, white clover (*T. repens*), and *M. truncatula* based on comparing DNA markers and their location in the genomes (Isobe et al., 2012). Our results are supported, too, by a recently published paper regarding WGS of red clover and construction of its physical map (De Vega et al., 2015). A slight discrepancies in locations of homologous sequences are likely the result of different methodology compared to previously published papers (Isobe et al., 2012; De Vega et al., 2015).

### DNA markers

DNA markers have a broad spectrum of uses in both research and practical breeding. They are used in QTL mapping (Zhao et al., 2013), evolution relationship studies (Ghamkhar et al., 2012; Isobe et al., 2012), variability assessment and genotyping of breeding material (Younas et al., 2012; Cidade et al., 2013), marker-assisted selection, and even gene pyramiding (Qi et al., 2015). Based on NGS technology, we are capable of discovering thousands of SSR and many millions of SNP markers (Zalapa et al., 2012).

Searching for SSR loci within 64,761 predicted coding sequences resulted in the identification of 6,749 SSR loci. This is more than twice the number of SSR loci identified from red clover transcriptome sequencing (Yates et al., 2014). On the other hand, due to the lower number of genes and shorter length of coding sequence described by RNA sequencing, the average SSR marker frequency is very similar (1 SSR marker per 13.42 kbp; Yates et al., 2014). When multiple repeat occurrences are taken into account, 10.4% of genes on average contained an SSR locus. This frequency is comparable to that reported in *Prosopis alba* (11%; Torales et al., 2013) but lower than those in *Nothofagus nervosa* (15%; Torales et al., 2012) and oak (19%; Ueno et al., 2010 and 24%; Durand et al., 2010). In coding sequences, clear domination of trimeric motifs (78.68%) is observed when compared to the SSRs from the genome as a whole (26.9%; Ištvánek et al., 2014). Much lower frequencies were found for other motifs. Similar results have been obtained also in other species, such as *N. nervosa* (Torales et al., 2012) and oak (Ueno et al., 2010). This phenomenon is very likely connected to the need to preserve open reading frame within the coding sequences and negative selection pressure against those SSR loci breaking it. Even in the majority of SSR loci with non-trimeric basic motifs, therefore, the combination of motif length and its repeat number is divisible by three—e.g., (A)12, (GA)6, (ATTGG)3—and this, then, does not violate reading frame by frame-shift mutations (Metzgar et al., 2000).

SNPs were also identified in coding sequences of red clover. Their greater frequency throughout the genome (343,027 SNP markers; 1 SNP per 144.6 bp) makes them useful in such high-throughput methods as SNP arrays (Víquez-Zamora et al., 2013; Yu et al., 2014). Nevertheless, when compared to other plant species, it is clear that SNP frequency is influenced by many factors, such as the number of individual plants analyzed in the study, natural variability in the population of the studied species, etc. In *P. alba*, for example, 1 SNP marker was found for every 2,512 bp (Torales et al., 2013), in *Capsicum annuum* 1 for every 2,253 bp (Ashrafi et al., 2012), in oak 1 for every 471 bp (Ueno et al., 2010), and in *Eucalyptus grandis* 1 for every 192 bp (Novaes et al., 2008). In these studies, the SNP number found correlated mainly with the number of individuals analyzed (e.g., 21 individual plants in oak, more than 200 in *E. grandis*). Although, just 16 individual plants of the same variety were analyzed in red clover, even higher SNP frequency was obtained, likely due to the outcrossing nature of clovers. Within the identified SNPs, transitions (63.52%) showed significant dominance over transversions (35.29%). These results are consistent with those in *P. alba* (Torales et al., 2013) and *Cucurbita pepo* (Blanca et al., 2011). A large number of SNPs are now available in red clover for genome-wide association studies and SNP microarray construction, where tens of thousands of markers are required.

### Validation of DNA markers and polymorphism

Outcrossing species populations are exceptionally variable and with a high level of heterozygosity. The majority of genetic analyses of such species are necessarily carried out in pooled samples in order to collect most of the population variability and also minimize costs. Results obtained from such pooled samples are, however, unsuitable for estimating the copy number of individual alleles, which precludes assessment of exact allele frequencies required to calculate polymorphic information content (PIC; Botstein et al., 1980). Recent advances in NGS technologies enable determination of allele frequencies from pooled samples (Mullen et al., 2012; Lynch et al., 2014), but these are very expensive and thus inaccessible especially for breeders working with non-model crops.

PIC is commonly used in plant genetics to assess polymorphism level for a marker locus. For leguminous plants, PIC was recently used, for example, for evaluating 48 SSR markers of *Vigna radiata* (Shrivastava et al., 2014), 45 SSR markers of *Trifolium alexandrinum* (Verma et al., 2015), and 36 SSR markers of *Vicia* spp. (Raveendar et al., 2015). Estimated PIC is usually directly connected with suitability for subsequent utilization, such as in variety identification or selection of suitable material for breeding purposes. In order to calculate PIC, a precise determination of allelic frequencies in the studied population is required.

To overcome the disadvantages of pooled samples, we proposed a modified PIC value termed pooled polymorphic information content (pPIC) which does not rely on determining allelic frequencies in the selected population. pPIC ranges from 0 to 1, similarly as does PIC, and it estimates the probability that two randomly collected pooled samples from the given species will differ in the given marker. Unlike PIC, pPIC works well for assessing the polymorphism level of a marker locus for pooled samples. The presented pPIC of the 95 SSR markers analyzed should, however, be taken into account only to evaluate pooled samples similar in size to that of our study. A significant decrease or increase in pooled sample size could shift pPIC and thus degrade the estimation of SSR marker discrimination power. Our results based on a pooled sample size of 16 plants should nevertheless be optimal for most potential subsequent utilizations. This is particularly important for screening gene bank accessions and large-scale analysis of cultivar identity and seed purity. For red clover, moreover, the optimal bulk size for genetic variation assessment among cultivars has been determined as 20 (Kongkiatngam et al., 1996).

This study generated a collection of 22 highly polymorphic SSRs with pPIC >0.9 and thus primer pairs for application to diversity studies in *T. pratense*. Seventy-two SSRs out of 95 validated showed pPIC >0.5. The single SSR marker in coding sequences SSR-TP_g20700.t1.cds3 was amplified in all 50 varieties but was also monomorphic (pPIC = 0.0). All other SSRs revealed some polymorphism in the analyzed variety populations. For the validated SSRs, the actual length range of amplified fragments corresponded with expectations with the single exception of SSR-TP_g32548.t1.cds1, whose amplified fragment was shorter (100–200 bp) than expected (237 bp).

The breeding methods in red clover include procedures suitable for outcrossing crops. Useful variation in a breeding population can be generated through hybridization and genome introgression, or by chromosome doubling (polyploidy) by colchicine. Subsequent phenotypic selection of superior individual plants or mass selection must be conducted on the progeny combining the best traits, and successive population breeding is performed. Molecular characterization of the analyzed varieties using SSRs reflects their genetic relationships, and the grouping is shown in Figure 7 and Figure S1. Tracing the breeding history revealed frequent sharing and exchange of cultivars and newly bred materials among European breeding stations. It was shown that varieties from sub-clusters IA, IIA, IIB1, and IIB2-1 had higher relatedness than varieties from sub-cluster IIB2-2. The possible reasons could be (i) introgressions from landraces and (ii) that the varieties were mostly released from 1970 to 1990, Chlumecky as early as 1935 (with the exceptions of Atlantis and Slavoj). Narrowing of the genetic base in the more recent varieties in sub-cluster IIB2-2 was also apparent.

The SSR profiling (i) differentiated varieties with possible introgressions from landraces and (ii) indicated the existence of diversity at the molecular level among different red clover varieties. The finding of inter-variety heterogeneity has important consequences for breeders who use these varieties. Cluster analysis by means of DNA profiling using the validated SSR set is suitable for such study.

Further progress in red clover breeding can be made by crosses with more distant genotypes as sources of new genetic variability, with new introgressions of important loci for resistance and quality. The identification of SSR or SNP markers in known-function genes linked to specific traits can facilitate marker-assisted selection. One important task is to develop a platform for red clover genotyping, employing genome-wide distributed SNP markers. The Tatra-derived reference sequence was initially used for the detection of the predicted 343 thousand SNPs. We used a preliminary set of 8,623 genome-wide distributed SNPs for polymorphism evaluation in individual plants. Arrayit methods provide universal microarray-based platforms for SNP genotyping (Schena et al., 1996). Sixteen of the validated SNPs were monomorphic and 8,607 were polymorphic with a mean PIC of 0.338. SNP validation confirmed the high quality of SNPs chosen for microarray. More sequenced red clover varieties/genotypes and a large set of informative SNPs are greatly needed for genotyping and association study. NGS methods such as genotyping-by-sequencing and the resequencing of targeted DNA regions from contrasting genotypes appear to be the most essential for SNP discovery and genotyping applications in red clover breeding. Temperature switch PCR can be successfully used in diagnostic applications through single-marker SNP genotyping for targeted coding sequences and for heterozygosity or homozygosity confirmation in validated loci. Large SNP sets are already available in grain legumes such as soybean (Song et al., 2013; Lee et al., 2015) and pea (Sindhu et al., 2014; Tayeh et al., 2015), or in peanut (Pandey et al., 2017). High-density SNP microarrays can significantly advance breeding applications.

## Author contributions

JI, JN, and JŘ designed the study. JI processed sequencing data, characterized protein-coding genes, and collaboratively with JD performed detailed inspection of gene annotation manually. JI performed gene classification into metabolic and biosynthetic pathways, comparison with other legumes, and generated genome-wide SSR and SNP markers. LP and JD prepared biological material, performed DNA isolation and marker validation. JD and PD performed pPIC and PIC calculation, polymorphism evaluation and phylogenetic analysis. JR supervised all aspects of the presented analyses. All of the authors contributed to the writing of the manuscript.

### Conflict of interest statement

The authors declare that the research was conducted in the absence of any commercial or financial relationships that could be construed as a potential conflict of interest.
