# Supplementary material for: Gene Classification and Mining of Molecular Markers Useful in Red Clover (Trifolium pratense) Breeding
Source: Front Plant Sci. 2017 Mar 22;8:367. doi: 10.3389/fpls.2017.00367 (PMC5360756; doi:10.3389/fpls.2017.00367)
Supplement: Supplementary file 10 [file Image1.PDF]

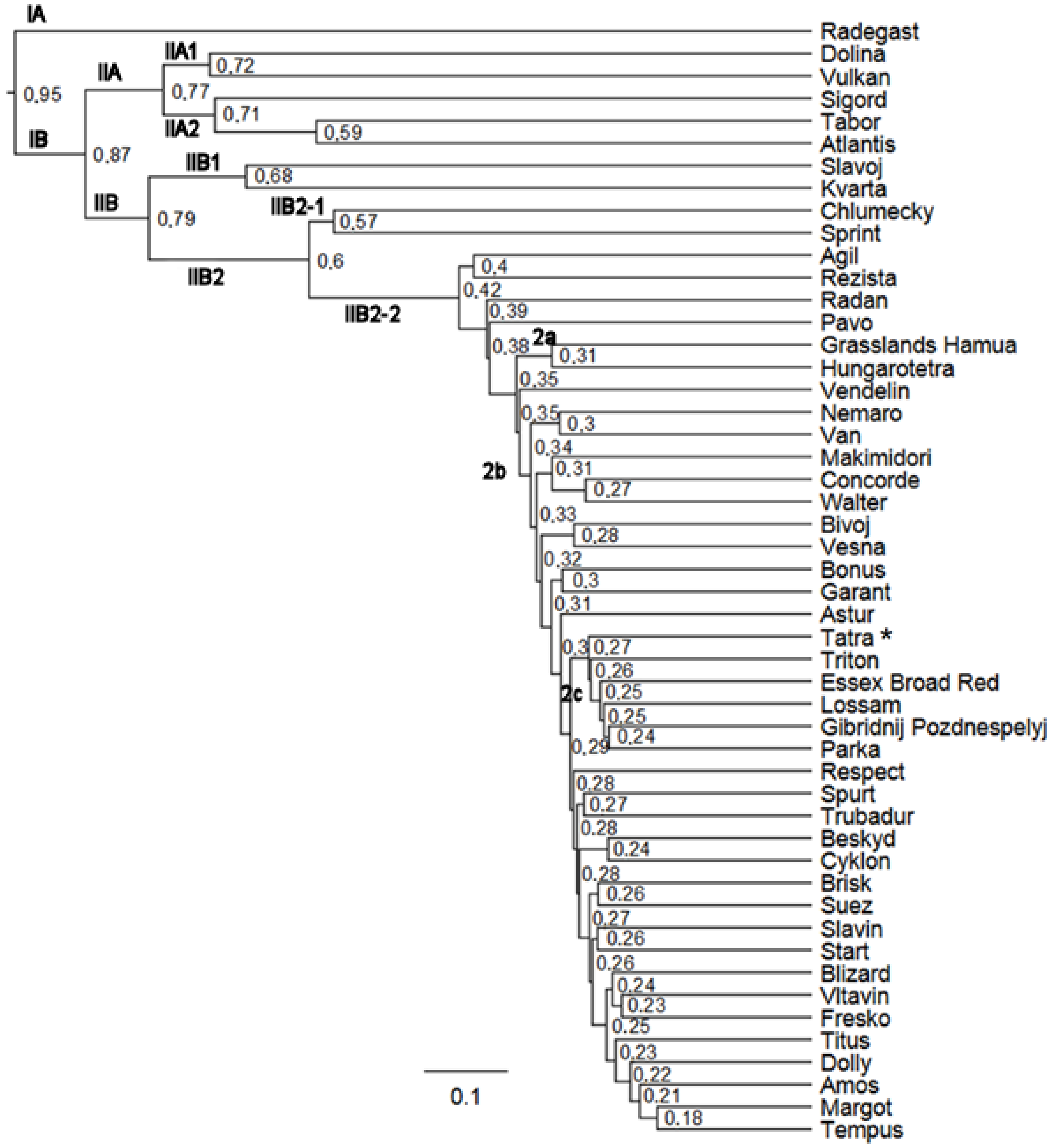

Figure S1. Study of red clover variety divergence using SSR markers (Jaccard index).
*** reference genotype**
